# Esculetin Alleviates Nonalcoholic Fatty Liver Disease on High-Cholesterol-Diet-Induced Larval Zebrafish and FFA-Induced BRL-3A Hepatocyte

**DOI:** 10.3390/ijms24021593

**Published:** 2023-01-13

**Authors:** Ji Ma, Yang Deng, Tingting Yang, Maoru Li, Jing Shang

**Affiliations:** School of Traditional Chinese Pharmacy, Jiangsu Key Laboratory of TCM Evaluation and Translation Research, China Pharmaceutical University, Nanjing 211198, China

**Keywords:** esculetin, non-alcoholic fatty liver disease, lipid-lowering, antioxidant

## Abstract

Non-alcoholic fatty liver disease (NAFLD), defined in recent years as metabolic-associated fatty liver disease (MAFLD), is one of the most common liver diseases in the world, with no drugs on market. Esculetin (ESC) is an active compound discovered in a variety of natural products that modulates a wide range of metabolic diseases and is a potential drug for the treatment of NAFLD. In this study, we used an HCD-induced NAFLD larval zebrafish model in vivo and an FFA-induced BRL-3A hepatocyte model in vitro to evaluate the anti-NAFLD effect of ESC. Lipid lowering, anti-oxidation and anti-inflammation effects were revealed on ESC and related gene changes were observed. This study provides a reference for further study and development of ESC as a potential anti-NAFLD/MAFLD drug.

## 1. Introduction

Since nonalcoholic fatty liver disease (NAFLD) and nonalcoholic steatohepatitis (NASH) were first described in the 1990s, they have gone from obscure liver diseases to the most prominent cause of chronic liver disease with a 25% prevalence worldwide [1]. NAFLD involves a spectrum of lesions ranging from hepatic steatosis to NASH, is characterized by hepatocyte injury, apoptosis, cell death and inflammation, and further deteriorates into fibrosis and cirrhosis [2]. Recently, an expert group proposed changing the term “NAFLD” to a new definition, MAFLD (metabolic-related fatty liver disease), considering the primary role of metabolic dysfunction [3,4]. However, due to the lack of comprehensive cognition of the multiple pathogenesis factors of NAFLD/MAFLD, there are still no Food and Drug Administration-approved drugs on the market for NAFLD.

The pathogenesis of NAFLD is complex. A classic hypothesis, the “Two hits” theory, elucidates the pathogenesis of NAFLD in two respects: hepatic overlord-lipid accumulation and oxidant stress [5,6]. Animal and clinical data indicate that many cases of hepatic steatosis do not develop necrotizing inflammation or fibrosis. However, lipid peroxidation is the mechanism that links the hepatic steatosis to necrotizing inflammation and fibrosis and is the “second hit” in the “two hits” theory [6]. As new research develops and pathologists gain new insights into NAFLD, the “second hit” theory is no longer sufficient to explain the complex pathogenesis of NAFLD. The new concept of “multiple hits” has emerged, involving insulin antagonism, lipid toxicity, oxidative stress, inflammation, liposomes, bile acid, etc. [7]. What both theories have in common is a recognition of the important role of lipid oxidation and inflammation. Therefore, antioxidant and anti-inflammatory therapies should be considered beneficial for NAFLD.

The Zebrafish *(Danio rerio*) genome is 87% similar to that of human beings and has become a widely used disease model in multiple fields in recent years [8,9,10]. NAFLD Larval zebrafish model was comprehensively studied several years ago [11], and been widely used as a research model or screen model in studying anti-NAFLD drugs [12,13]. The test of pathology, biochemistry index and genome expression changes in NAFLD has been well established in larval zebrafish for compound screening, and the features of low-dose and low-time consumption make it a viable option for active compounds.

Esculetin (ESC) is one of the active compounds discovered from multiple nature products, such as *Cortex Fraxini*, *Citrus limon Osbeck*, *Euphorbia lathyris*, *Artemisia capillaris* and *Cichorium glandulosum Boiss et Huet*. ESC has been reported take the effects on reversing metabolic related disorder [14]. Some researchers have studied the effects of ESC on insulin resistance in diabetic model animals and found that ESC increases insulin sensitivity [15]. Other researchers examined the liver preservation effects of ESC in animal models of acute liver injury and found that ESC improves liver fibrosis through mechanisms related to energy metabolism and lipid pathways [15]. In addition, ESC improves oxidative stress levels and immune balance [16]. Since many reports have claimed that ESC takes the effect on various disease with multiple mechanism related to NAFLD, however, the specific study of whether ESC could prevent NAFLD effectively is still lacking. 

In this study, we used NAFLD larvae zebrafish model in vivo and the hepatocyte model in vitro to investigate the potential role of ESC ameliorating NAFLD.

## 2. Results

### 2.1. Lipid Lowering Effect of ESC on an HCD-Induced Larval Zebrafish Model

**Figure 1:**
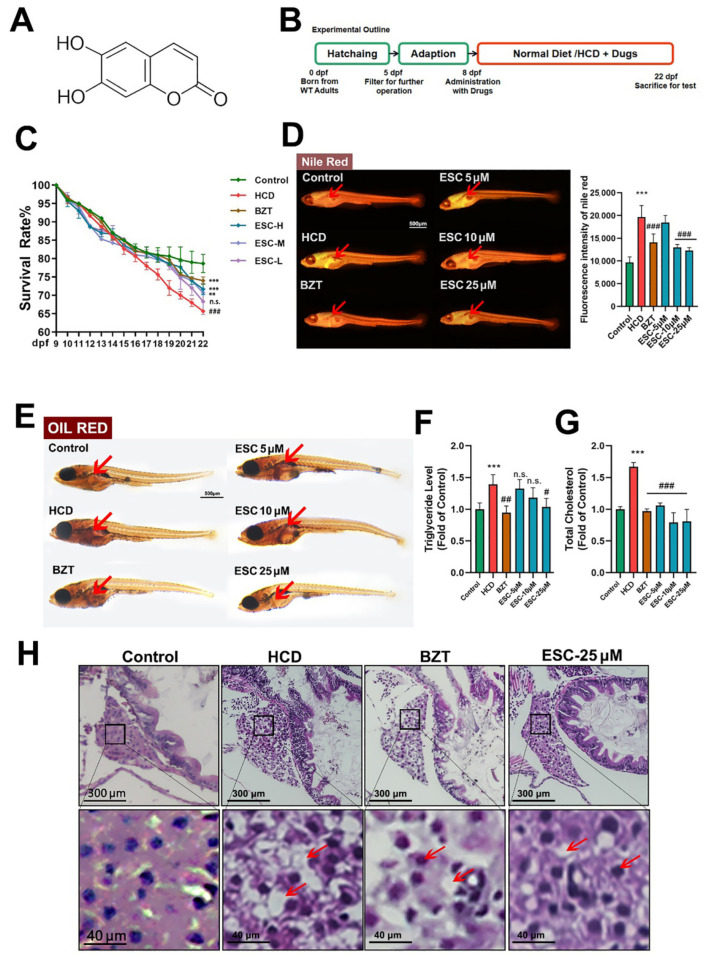
Lipid lowering effect of ESC on an HCD-induced larval zebrafish model. (**A**) The chemical structure of ESC. (**B**) Experimental outline of the larval zebrafish experiments. (**C**) Survival rate of Tablelarval zebrafish. (**D**) Nile red stain of larval zebrafish. (**E**) Oli red stain of larval zebrafish. (**F**) Triglyceride of larval zebrafish. (**G**) Total cholesterol of larval zebrafish. (**H**) HE stain of larval zebrafish liver, and the hepatic steatosis is pointed by red arrows. Bar indicates means ± SD. ** *p* < 0.01, *** *p* < 0.001 represent compared with the control; n.s. represents no significance; # *p* < 0.05, ## *p* < 0.01, ### *p* < 0.001 represents compared with the model. *p* < 0.05 was considered as statistically significant calculated by One-way ANOVA followed by Tukey’s test (*n* = 3, n indicates the replicates of the experiment).

The >99% purity esculetin (Figure 1A) was used for the experiment. To investigate the role of ESC in regulating lipid metabolism, a NAFL larval zebrafish models was established (Figure 1B). Benzabate (BZT, 10 μM/L) was used as positive drug, and three doses (ESC-L 5 μM/L, ESC-M 10 μM/L and ESC-H 25 μM/L) of ESC were established for further test. The survival rate of each group of zebrafish was recorded (Figure 1C). Significantly, compared with HCD group, the survival rate in BZT, ESC-H and ESC-M groups was reversed to the control group. The lipid accumulation in whole fish was stained by Nile red (Figure 1D), and fluorescence intensity of each fish were quantified. Obviously, the lipid accumulated in abdomen of fish (point by red arrows), and lower fluorescence intensity can be observed in all drugs groups. Further oil-red staining showed that both BZT and ESC reduced liver steatosis (Figure 1E). Lipid levels of TG and TC were measured with kit and both ESC and BZT groups were found to have a significant lipid reduction effect on TG and TC (Figure 1F,G). In the HE staining, macrovesicular steatosis was observed in hepatic cells of the HCD group; however, macrovesicular steatosis in the BZT and ESC-H group was less observed in hepatic cells (Figure 1H). The results showed that the ESC had a lipid lowering effect on NAFL larval zebrafish model, and multiple staining confirmed that ESC reversed the hepatic steatosis. 

### 2.2. Anti-Oxidant Effect of ESC on an HCD-Induced Larval Zebrafish Model

**Figure 2:**
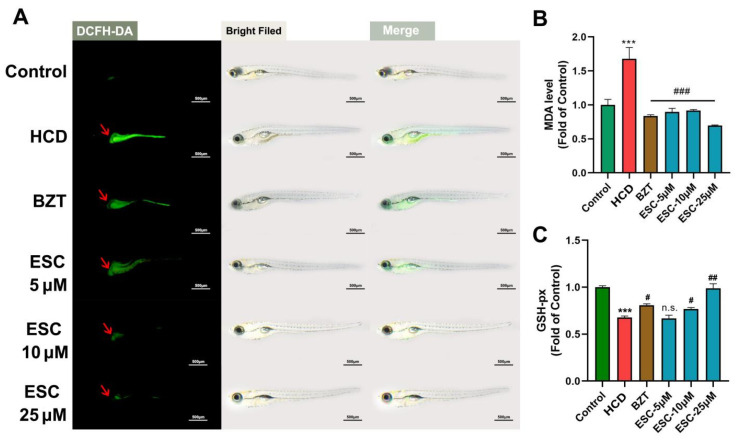
Anti-oxidant effect of ESC on an HCD-induced larval zebrafish model. (**A**) The DCFH-DA stain of larval zebrafish. (**B**) MDA of larval zebrafish. (**C**) GSH-px of larval zebrafish. Bar indicates means ± SD. *** *p* < 0.001 represents compared with the control; n.s. represents no significance; # *p* < 0.05, ## *p* < 0.01, ### *p* < 0.001 represents compared with the model. *p* < 0.05 was considered as statistically significant calculated by One-way ANOVA followed by Tukey’s test (*n* = 3, n indicates the replicates of the experiment).

As the lipid peroxidation plays a key role in the “second hits” of NAFLD, we investigated the anti-oxidant effect of ESC on the HCD-induced larval zebrafish model. A fluorescence probe DCFH-DA was used to detect the ROS in vivo of larval zebrafish (Figure 2A). Compared with the control group, a higher fluorescence was observed in the abdomen of fish (point by red arrows) of the HCD group, where existed the same area of lipid accumulation shown in Figure 1D. However, ESC decreased the fluorescence of ROS dose-dependently compared with the HCD group. From the test results of MDA (Figure 2B), an end product of lipid peroxidation, ESC showed a significantly lowering effect on MDA. Additionally, the test of glutathione peroxidase indicated that ESC may take the effect of an antioxidant by increasing the antioxidizing system (Figure 2C).

### 2.3. NAFLD Related mRNA Expression Changes in ESC on the HCD-Induced Larval Zebrafish Model

**Figure 3:**
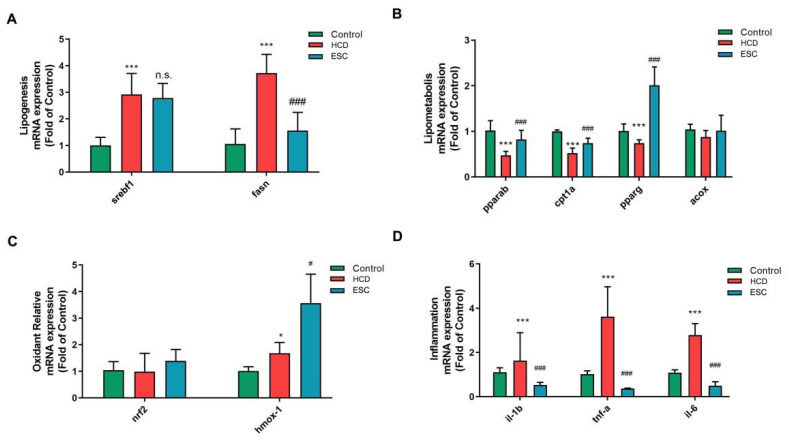
NAFLD-related mRNA expression changes in ESC on the HCD-induced Larval Zebrafish Model. (**A**) The gene expression level of lipogenesis on larval zebrafish. (**B**) The gene expression level of lipometabolism on larval zebrafish. (**C**) The gene expression level of oxidant stress on larval zebrafish. (**D**) The gene expression level of inflammation on larval zebrafish. Bar indicates means ± SD. * *p* < 0.05, *** *p* < 0.001 represent compared with the control; n.s. represents no significance; # *p* < 0.05, ### *p* < 0.001 represents compared with the model. *p* < 0.05 was considered as statistically significant calculated by One-way ANOVA followed by Tukey’s test (*n* = 3, indicates the replicates of the experiment).

The previous results confirmed that ESC could take an anti-NAFLD effect by lipid lowering and antioxidant on the larval zebrafish model. To further reveal the mechanism of ESC on multiple pathogenesis aspects of NAFLD, we performed a real-time quantitative polymerase chain reaction (RT-QPCR) experiment to detect the mRNA expression changes in lipogenesis, lipid metabolism, oxidation stress, fibrosis, and inflammation. As shown in Figure 3A, fatty acid synthase (*fasn*) mRNA expression decreased in the ESC group compared with the model group. However, there was no significant change in the expression of *serbf1*, a sterol regulatory element-binding factor. As for lipometabolism-related mRNA expression (Figure 3B), the peroxisome proliferator-activated receptor alpha (*ppara*), recombinant carnitine palmitoyl-transferase 1A (*cpt1a*) and peroxisome proliferator-activated receptor gamma *(pparg*) significantly increased in the ESC group compared with the model group. In addition, the expression of the acyl-coA oxidase (*acox*) changes was not significant. These results reveal the potential lipid regulatory mechanisms of ESC.

In terms of oxidative stress-related genes, mRNA expression of nuclear factor-like 2 (*nrf2*) was not significantly changed. However, heme oxygenase 1 (*hmox-1*) expression was significantly higher in the ESC group than in HCD group (Figure 3C). The results indicated that HO1 related pathway is key to the reduction of ROS effects in ESC. To inflammatory gene expression, the expression levels of interleukin-1 beta (*il-1b*), tumor necrosis factor alpha (*tnf-a*), and interleukin-1 (*il-6*) were significantly lower in the ESC group than in the HCD group. 

### 2.4. Effects of ESC on FFA-Induced BRL-3A Hepatocyte In Vitro

**Figure 4:**
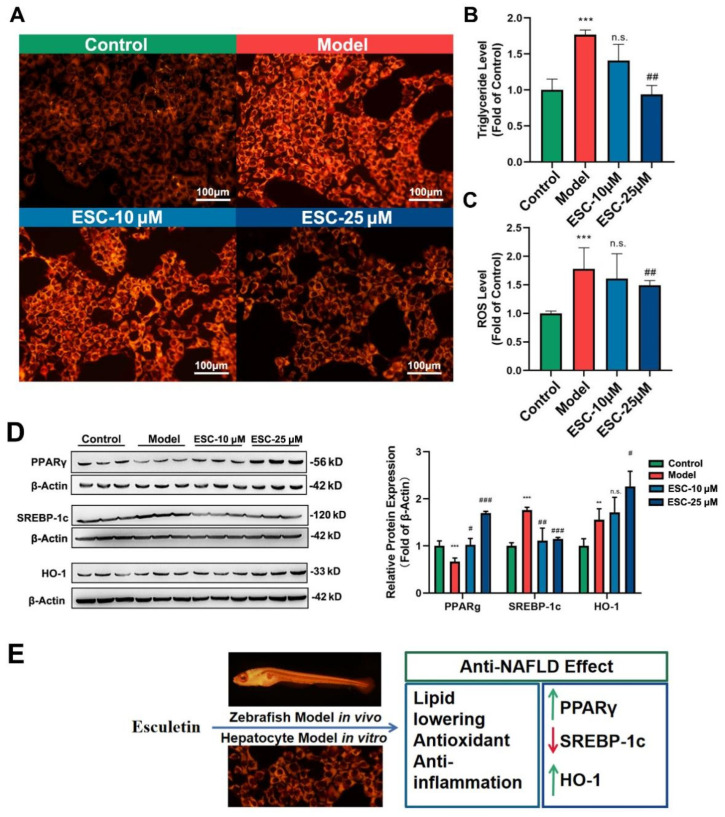
Effects of ESC on FFA−induced BRL−3A hepatocyte *in vitro*. (**A**) The BRL−3A hepatocyte stained with nile red. (**B**) The Triglyceride (TG) level of BRL-3A hepatocyte. (**C**) The ROS level of BRL−3A hepatocyte. (**D**) Western blot and relative protein expression of BRL−3A hepatocyte. (**E**) Summary of the study. Bar indicates means ± SD. ** *p* < 0.01, *** *p* < 0.001 represents compared with the control; n.s. represents no significance; # *p* < 0.05, ## *p* < 0.01, ### *p* < 0.001 represents compared with the model. *p* < 0.05 was considered as statistically significant calculated by One-way ANOVA followed by Tukey’s test (*n* = 3, indicates the replicates of the experiment).

Abundant results in vivo showed the anti-NAFLD effect of ESC on lipid lowering and antioxidants, and multiple mRNA expression indicated the potential mechanism of ESC on reducing lipid accumulation, antioxidant activity, and inflammatory response; however, a direct effect of ESC on the hepatic cells still needs to be investigated. A FFA-induced BRL-3A model was established and ESC was treated with high (ESC-H 25 μM) and low (ESC-L 10 μM) doses. In terms of the staining of Nile red shown in Figure 4A, from the fluorescence picture we can see that ESC reduced fluorescence intensity significantly in the 25 μM group. TG and ROS level (Figure 4B,C) were both decreased in the ESC-25 μM group compared with the model group. Further results from WB (Figure 4D) showed that ESC increased PPARγ and HO-1 protein expression and decreased the SREBP-1c protein expression.

## 3. Discussion

Though the pathogenesis of NAFLD is complex and still revealing in multiple novel aspects, the name of NAFLD even changed for its uncertain pathogenesis to MAFLD; lipid metabolism disorder and oxidative are the two key factors in the time of NAFLD or the new time of MAFLD [7]. Results from larval zebrafish in vivo and BRL-3A hepatocyte in vitro showed the specific lipid lowering and antioxidant effect of ESC on NAFLD, suggesting that ESC may be a potential therapeutic agent for further development of NAFLD/MAFLD. However, as a novel active compound extracted from natural plants, the production manufacturer of ESC still needs further development. NAFLD larval zebrafish is a novel in vivo screen model for compounds that are hard to produce [11], demonstrating a systematic study for ESC study in vivo, and providing reference data for further pr-clinical study on a classical rodent model for ESC.

PPARγ is one of the peroxisome proliferator-activated receptors (PPAR) and a member of the nuclear receptor transcription factor superfamily that regulates the expression of target genes related to energy regulation [17]. PPARγ regulates the pathophysiological process in various diseases related to metabolism and inflammation, and is famous as a target of insulin sensitizer thiazolidinedione drugs (TZDs) [18]. PPARγ is proved to be closely associated with hepatic lipid metabolism and play important roles in NAFLD [19] The hepatic PPARγ plays a role in the development of fatty liver in the NAFLD patients. Hepatic PPARγ independently regulates the liver lipid accumulation in mice, and in recent years PPAR-γ expression has been identified as an additional signaling that modulates the SREBP-1c to trigger hepatic steatosis. As the results showed, ESC promoted PPARγ gene expression in vivo and protein expression in vitro. This indicates that PPARγ may be a key regulator for ESC in regulating lipid metabolism. Sterol regulatory element-binding protein-1c is a key lipogenic transcription factor activated by insulin. SREBP1c is considered to be a master regulator of hepatic lipogenesis; it has the ability to regulate lipogenic gene expression, fatty acid, and TG homeostasis. In addition, the role of SREBP-1c in de novo lipogenesis and NAFLD pathogenesis has been widely recognized and makes it a potential therapeutic target for the treatment of NAFLD [20,21]. Meanwhile, the specific lipid synthesis regulation gene *fasn* and the protein of SREBP-1c were decreased by ESC in vivo and in vitro, which further explain the mechanism of ESC on lipid regulation. Moreover, ESC increased gene expression of *homox-1* and protein expression of HO-1, a downstream target of Nrf2 with antioxidant and anti-inflammation effect [22], clarify the mechanism of ESC on lowering ROS and increasing GSH-px, which is the mechanism of antioxidant on ESC.

## 4. Material and Methods

### 4.1. Reagents and Solution

Esculetin was purchased from Aladdin (Shanghai, China), the purity of which was above 99%. Aladdin (Shanghai, China) provided the Dihydroethidium (95%), Nile red (95%), Bezafibrate (98%), and Oil red (98%). Cholesterol and 2,7-Dichlorodi-hydrofluorescein diacetate (DCFH-DA, 95%) were from Sigma-Aldrich (St. Louis, MO, USA).

### 4.2. Maintenance of Zebrafish and Treatment

Wild-type AB-line zebrafish was reared in filtered circulating water with a light cycle of 14:10 (light: dark) hours at 28.5 °C. Embryos were produced naturally using wild-type AB-line maturated zebrafish. Zebrafish embryos grew freely in egg water between 28.5 °C and 5 days after fertilization, with a light cycle of 14:10 (light: dark) hours (dpf). Larval zebrafish were then randomly divided into six groups (*n* = 100 for each group).

In the administration of zebrafish, 5% cholesterol-diet (HCD) basic food of larval zebrafish (Zeigler larval AP100, Gardners, PA, USA) was prepared as previously reported [11]. ESC and Bezafibrate (BZT) were dissolved in DMSO, and diluted with water to the final working concentration of ESC (5 μM, 10 μM, and 25 μM) and BZT (10 μM). 

HCD was given from 6dpf. The treatment groups were treated with drugs from 8dpf, while the control group was fed a standard diet. The experiment was stopped at 21dpf. All groups performed maintenance according to the schedule shown in Figure 1B. Additionally, this zebrafish model was believed to be a nonalcoholic fatty liver (NAFL) model for further study. All the animal experiments was approved by Ethical Committee of China Pharmaceutical University (SYXK(SU)2021-0010), and Laboratory Animal Management Committee of Jiangsu Province. All the experiment was followed the Jiangsu Provincial standard ethical guidelines in using experimental animals under the ethical committees mentioned above.

### 4.3. Oil Red Staining and Histopathology

Zebrafish were anesthetized by using 0.05% tricaine before full staining. We dissolved 0.5 mg/mL oil red with isopropyl alcohol and diluted with water (3:2, *v:v*) to produce the oil red working liquid. Furthermore, the oil red working solution was filtered until the supernatant was clear and transparent. Meanwhile, 4% paraformaldehyde-fixed zebrafish were stored at 4 °C for 24 h prior to the experiment. Samples were washed 3 times and then soaked for 5 s in isopropyl alcohol. After sample preparation, the sample was dyed with the oily red working liquid. The zebrafish samples were stained for 1 h. The zebrafish blocks were sliced to 4 μm for HE staining. Photos were taken in stereoscope (Olympus SZX16, Olympus, Tokyo, Japan).

### 4.4. Fluorescent Staining and Quantification

Nile red stains and DCFH-DA stains were used in our experiments. Nile red is a kind of photo-stable lipophilic dye with bright red fluorescence, commonly used in neutral lipid staining. Nile red has an excitation wavelength of 543 nm and scattering wavelength of 598 nm. DCFH-DA is a cell-permeable probe used to detect reactive oxygen species (ROS). ROS converts non-fluorescent samples to fluorescent samples by producing dichlorofluorescence (DCF) in living cells at excitation and scattering wavelengths of 480 nm and 525 nm, respectively. 

In the trial, zebrafish were maintained in darkness at 28.5 °C for 30 min at 0.5 μg/mL Nile red or 10 μM/L DCFH-DA. Zebrafish were dyed, washed three times with egg water, anesthetized with 0.05% tricaine, and then immobilized with 4% CMC-Na. Nile red or DCFH-DA stained images were observed and taken using a fluorescent stereoscope (Olympus SZX16). The exposure intensity and time corresponding to the same stain were consistent for comparison. 

### 4.5. Biochemical Measurement

Each group contains three samples, including six zebrafishes; 0.05% tricaine was euthanized and biochemical samples were shredded with ultrasound. Levels of triglyceride (TG), total cholesterol (TC), malondialdehyde (MDA), and GSH-px were measured using commercial assay kits (Jiancheng, Nanjing, China) as per the manufacturers’ instructions.

### 4.6. Real-Time Quantitative PCR Analysis

A real-time RT-qPCR was used to detect expression of genes involved lipid metabolism, oxidation, and inflammation. Each group collected 20 larval zebrafish and euthanized them with Trizol reagent to extract total RNA. A reverse transcription kit was used the cDNA collecting process (PrimeScript RT Master Mix, Takara, Japan). Corresponding mRNA expressions was quantified using qPCR reagent (SYBR Green, Takara, Japan). All steps of the experiment were carried out in accordance with the manufacturers’ protocol in the kit. Oligo in QPCR was purchased from Genscript (Nanjing, China) as shown in Table 1. Expression levels of each target mRNA were calculated by normalizing the 2^−ΔΔCt^ method to GAPDH.

### 4.7. BRL-3A Cell Culture and Treatments

Rattus norvegicus hepatocellular cell line BRL-3A (American Type Culture Collection, Manassas, VA, USA) was cultured at 37 °C in DMEM supplemented with 10% fetal bovine serum (FBS) and 1% penicillin-streptomycin and in an atmosphere of 5% CO_2_. BRL-3A cells (1 × 10^6^ in number) were seeded in 100 mm plates until 70% confluent. The model group of BRL-3A cells was treated with 1mM FFA for 24 h. The H-ESC group was treated with 1Mm FFA combination with 25 μM ESC for 24 h. The L-ESC group was treated with 1mM FFA combination with 10 μM ESC for 24 h. The control group was treated with BSA and NaOH at final concentrations of 1% and 0.2 μM, respectively. 

### 4.8. Western Blotting

The cells were digested, centrifuged at 1200× *g* for 5 min, the supernatant was discarded, resuspended in RIPA buffer containing protease inhibitors, and mixed. The supernatant was isolated, assayed for protein content, and frozen at −80 °C at room temperature until use. Aliquots of supernatants containing 40 µg of protein were measured and mixed with 1/5 × Laemmle sample buffer (60 mM Tris-HCl pH 6.8, 25% glycerol, 2% sodium dodecyl sulfate, 14.4 mM β-mercaptoethanol and 0.1 % bromophenol blue), mixed, and heat-denatured at 95 °C for 5 min). Protein samples were then separated on 10% SDS-PAGE and electroblotted onto nitrocellulose membranes. After blocking with 5% skim milk in TBS buffer (20 mM Tris–HCl, 150 mM NaCl, pH 7.4), the membranes were incubated overnight at 4 °C prior to conferring antibodies. Antibodies included Anti-beta Actin [mAbcam 8226]—Loading Control (1:4000; #ab8226), recombinant Anti-PPAR gamma [EPR23297-111] (#ab272718), Anti-SREBP1 (#ab28481) and recombinant Anti-Heme Oxygenase 1 [EPR1390Y] (1:10,000, #ab68477) from Abcam, Cambridge, UK. Results were displayed with the ECL chemiluminescence detection kit (Perkinlemer, Waltham, MA, USA) and quantified with Image J software(Version 1.53).

### 4.9. Statistical Analysis

We applied Graph Pad PRISM (Version 8.02) (Graph Pad Software, San Diego, CA, USA) to analyze the data. Mean ± SD was presented in the form of all the data. One-way ANOVA was used in the significance calculation, and *p* < 0.05 in different groups was regarded as statistical significance.

## 5. Conclusions

In summary (Figure 4E), ESC took an anti-NAFLD effect by regulating lipid accumulation, anti-oxidation, and anti-inflammation in the HCD-induced NAFLD larval zebrafish model in vivo and FFA-induced BRL-3A hepatocyte model in vitro. Further gene and protein expression tests revealed that PPARγ, SREBP-1c, and HO-1 are key regulators of ESC on lipid-lowering and antioxidant activity. Further pharmacology study and mechanism research on mature models or clinical study could be performed after a mature production of ESC is established, and this study supplies a reference for ESC to be developed into a potential anti-NAFLD/MAFLD drug in the market.

## Figures and Tables

**Table 1:** Oligo in qPCR.

Gene Name	Species	Forward Primer (5′->3′)	Reverse Primer (3′->5′)	Tm (°C)
*serbf1*	*Danio rerio*	CCGGCTGCAGGTGTATGGAG	CTCGTGAGGCTAACCAGCGG	60.00
*fasn*	*Danio rerio*	AAGCGTGTTCGTGAGTGGCA	CAGGCCTCAGTGATGAGCCG	60.00
*pparβ*	*Danio rerio*	ACGGACGTATGCCAGAAGCG	TGACGTGCTTGGCCAGTGTT	60.00
*pparγ*	*Danio rerio*	ACATGCCGCTCCACGAGCAC	GTGGTCGGTCACCAGCTGCC	59.00
*cpt1a*	*Danio rerio*	TGGCACGGCTGGATGTTTGC	CATTGCGGGCACAAGCCCAT	58.45
*mmp9*	*Danio rerio*	TGCTTGACCAGCCCACCGTT	TCATTGCGGCCCTCACTGGT	58.33
*tgfb1*	*Danio rerio*	CACGGGAACGGGCAGCATGT	TCTGTCCGTCGTCGTCCGCT	59.97
*keap1*	*Danio rerio*	TGGCCAAAGCCTGCTGCGAT	AACCTCCACATGCCGCCACA	58.91
*nrf2*	*Danio rerio*	AGCGAACGCAGCAGCAACCT	TGGAGGCGCTCAAGCGCAAA	59.90
*il1b*	*Danio rerio*	AGCCGGAAGCAGCGACTTGA	ACGAGATGTGGAGCGGAGCCTT	58.72
*tnfa*	*Danio rerio*	AGCTTGAGAGTCGGGCGCTT	TGGCGGCACATTGCCAAGAGT	58.43
*il6*	*Danio rerio*	ACAGCCAGCTGCAGGTGAGAGA	ATGGCTCTGCAGGCGTCGAT	58.57

## Data Availability

The data used to support the findings of this study are available from the corresponding author upon request.
